# Bioactive Peptides from Yellowfin Tuna By-Products: Structural Characterization and Neuro-Related Activities in PC12 Cells

**DOI:** 10.3390/cimb48040374

**Published:** 2026-04-03

**Authors:** Yaqi Kong, Yifan Liu, Haoze Yang, Xianzhe Liang, Min Zhao, Ahsan Javed, Xiaozhen Diao, Wenhui Wu

**Affiliations:** 1 Department of Marine Pharmacology, College of Food Science and Technology, Shanghai Ocean University, Shanghai 201306, China; m230351113@st.shou.edu.cn (Y.K.); 2334214@st.shou.edu.cn (Y.L.); m230351143@st.shou.edu.cn (H.Y.); m240401215@st.shou.edu.cn (X.L.); m230300988@st.shou.edu.cn (M.Z.); ahsan@shou.edu.cn (A.J.); xzdiao@shou.edu.cn (X.D.); 2 Marine Biomedical Science and Technology Innovation Platform of Lin-gang Special Area, Shanghai 201306, China; 3 Department of Food Science, Government College University Faisalabad, Faisalabad 38000, Pakistan; 4 Putuo Sub-Center of International Joint Research Center for Marine Biological Sciences, Zhoushan 316104, China

**Keywords:** yellowfin tuna by-products, bioactive peptides, PC12 cells, neuro-related activity

## Abstract

Marine-derived bioactive peptides have attracted increasing attention as value-added functional ingredients. In this study, peptides (<3 kDa) were prepared from yellowfin tuna processing by-products and further fractionated by Sephadex G-25 gel filtration. The major fraction (TBP-MF) exhibited markedly improved compositional homogeneity compared with the unfractionated hydrolysate (TBP), providing a well-defined peptide system for subsequent characterization and biological evaluation. Physicochemical analyses demonstrated that TBP-MF possessed enhanced thermal stability and a more ordered secondary structure, characterized by pronounced β-sheet enrichment, as revealed by TGA/DSC, FTIR, and circular dichroism analyses. Morphological and colloidal characterization further showed that TBP-MF formed relatively uniform lamellar and fibrous assemblies with a narrower particle size distribution and reduced electrostatic stabilization, indicating a higher tendency toward ordered self-association. Peptidomic profiling combined with in silico analysis revealed that TBP-MF was enriched in short peptides with relatively higher PeptideRanker scores and a functional motif distribution containing relatively more neuro-related annotations, although angiotensin-converting enzyme (ACE)- and dipeptidyl peptidase IV (DPP-IV)-related motifs remained predominant in both groups. In differentiated PC12 cells, TBP-MF exhibited excellent cytocompatibility and induced a stable, concentration-dependent increase in the Cell Counting Kit-8 (CCK-8) readout (OD_450_), indicating enhanced cellular metabolic activity and/or increased cell number. In addition, TBP-MF significantly increased intracellular levels of key neurochemical factors associated with sleep-related regulation, including tetrahydrobiopterin (BH4), serotonin (5-HT), and γ-aminobutyric acid (GABA). Overall, this study highlights yellowfin tuna by-products as a promising marine resource for bioactive peptides and suggests that fractionation-driven structural refinement is associated with neuro-related biological activity in differentiated PC12 cells. These findings support the potential application of marine by-product-derived peptides as functional ingredients in health-related fields.

## 1. Introduction

Insomnia disorder imposes a substantial societal burden, yet current therapeutic options remain suboptimal [1]. While cognitive behavioral therapy for insomnia (CBT-I) is the first-line recommendation, limited accessibility often necessitates pharmacotherapy [2]. However, conventional sedative–hypnotics are associated with tolerance, dependence, cognitive impairment, and fall risks, particularly in long-term use [3,4]. Even alternatives like melatonin and orexin receptor antagonists show variable efficacy or inconsistent patient responses [5,6]. Consequently, there is a growing demand for safer, food-derived therapeutic strategies that can improve sleep quality without severe adverse effects.

Pathophysiologically, insomnia is closely linked to oxidative stress [7] and disrupted neurotransmitter homeostasis, particularly involving γ-aminobutyric acid (GABA) [8] and serotonin (5–HT) [7,8,9]. The biosynthesis of 5–HT is rate-limited by tryptophan hydroxylase 2 (TPH2), which strictly requires tetrahydrobiopterin (BH4) as a cofactor [10]. Crucially, intracellular BH4 levels are regulated by the enzyme GTP cyclohydrolase 1 (GCH1) and are highly susceptible to oxidative degradation [11,12]. Since oxidative stress depletes BH4—thereby throttling 5–HT synthesis—interventions that enhance GCH1 activity or protect BH4 via antioxidant mechanisms offer a precise neurochemical target for sleep support.

Food-derived bioactive peptides have emerged as promising candidates for such multi-target modulation [13]. Specifically, enzymatic hydrolysates from marine sources are gaining attention for their potential to cross the blood–brain barrier and exhibit neuroprotective activities [14,15,16]. Tuna dark-muscle by-products have been shown to yield low-molecular-mass peptides with notable antioxidant activity after enzymatic hydrolysis followed by Sephadex G-25 fractionation, supporting the feasibility of producing bioactive peptide fractions using workflows similar to those in the present study [17]. These observations suggest that tuna by-product-derived peptides may help support sleep-related neurochemical regulation through antioxidant and related bioactive effects, while also highlighting the potential valorization of fish processing by-products as high-value functional ingredients [13,17].

Despite this potential, direct evidence connecting tuna by-product-derived peptides to the specific GCH1–BH4–TPH2 sleep regulatory axis remains limited. Therefore, this study prepared a crude tuna blood–muscle peptide fraction (TBP) and an enriched major fraction (TBP–MF). We characterized their physicochemical properties and evaluated their regulatory effects on the BH4–TPH2–5–HT cascade in differentiated PC12 cells. The present study provides new insight into how marine-derived peptide fractions may support sleep-related neurochemical regulation through modulation of monoamine-related biosynthetic factors in differentiated PC12 cells.

## 2. Materials and Methods

### 2.1. Chemicals

Yellowfin tuna blood meat was provided by Zhejiang Rongchuang Food Industry Co., Ltd. (Zhoushan, China). Sodium hydroxide, hydrochloric acid, sodium chloride, and activated carbon were purchased from Sinopharm Chemical Reagent Co., Ltd. (Shanghai, China). Sephadex G-25 was acquired from Yuanye Biotechnology Ltd. (Shanghai, China). Trypsin (30,000 U/g) and Flavourzyme (a commercial flavor protease preparation derived from Aspergillus oryzae, 30,000 U/g) were purchased from Beijing Solarbio Technology Co., Ltd. (Beijing, China). N-butanol, absolute ethanol, methanol, and acetonitrile were procured from Titan Technology Ltd. (Shanghai, China). The TPH2 assay kit, GCH1 assay kit, GABA assay kit, BH4 assay kit, and 5–HT assay kit were all purchased from Jiangsu Meimian Industry Co., Ltd. (Yancheng, China). Unless otherwise specified, all chemical reagents utilized in the experimental group were of high analytical purity.

### 2.2. Preparation and Enrichment of Tuna By-Product Peptides

The raw material used was dark muscle (hemoglobin-rich tissue) collected from both sides of the backbone of yellowfin tuna, stored at −20 °C at the East Sea Marine Research Center [18]. The frozen tissue was thawed at room temperature, skinned, and deboned. The meat was rinsed with running water, then soaked in a 1.5% NaCl solution (1:5 *w*/*v*) overnight at 4 °C to remove residual blood. After draining, the meat was dried, crushed using a blender, and defatted by soaking in anhydrous ethanol for 6 h with continuous stirring. This defatting step was performed to minimize lipid interference and to ensure that subsequent fractionation and bioactivity evaluation primarily reflect the peptide fraction. The defatted residue was dried in an oven at 50 °C for 4 h to obtain minced fish powder, which was stored at –20 °C.

Fifty grams of the prepared tuna meat powder was mixed with pure water at a solid-to-liquid ratio of 1:9 (*w*/*v*) and thoroughly homogenized. The pH was adjusted to 8.0 using 1 M sodium hydroxide (NaOH). Trypsin (2%) was added, and hydrolysis was performed at 37 °C for 3 h [18]. Subsequently, the pH was adjusted to 7.0 using 1 M NaOH or 0.1 M HCl, and 1% Flavourzyme was added for further hydrolysis at 50 °C for 4 h. These hydrolysis conditions were selected based on preliminary single-factor experiments followed by response surface methodology (RSM) optimization, using degree of hydrolysis and antioxidant activity as practical response indicators. Under these criteria, the selected conditions provided a reproducible working optimum for subsequent fractionation and cellular evaluation. Upon completion, the enzymes were inactivated by heating at 100 °C for 15 min. The mixture was cooled to room temperature and centrifuged at 10,000 r/min for 20 min at 10 °C. The supernatant was collected, decolorized with 1% activated charcoal for 1 h, and centrifuged again. The resulting crude peptide solution was processed through an ultrafiltration centrifugal unit (MWCO = 3 kDa, 3000× *g*, 1 h) [19,20]. The filtrate (<3 kDa) was designated as the tuna blood-meat peptide fraction (TBP). The TBP was further fractionated using a Sephadex G-25 column, and the dominant, well-resolved peak fraction was collected to obtain the enriched major fraction (TBP-MF) [20,21]. Both fractions were lyophilized and stored for subsequent analysis.

### 2.3. Fractionation by Sephadex G-25 Gel Filtration Chromatography

The crude peptide solution was filtered through a 0.45 μm membrane filter and fractionated using a Sephadex G-25 column (2 cm × 50 cm) [21,22]. The column was pre-equilibrated with deionized water. A sample volume of 2 mL was loaded onto the column and eluted with deionized water at a flow rate of 0.5 mL/min at room temperature. The elution profile was continuously monitored at 220 nm using a UV detector (LC-UV310, Shanghai Wufeng Scientific Instruments Co., Ltd., Shanghai, China). The significant fractions were collected based on the chromatographic peaks, lyophilized, and stored for subsequent structural characterization and physicochemical analysis.

### 2.4. Thermal Property Analysis (TGA–DSC)

The thermal stability of the target protein peptides (TBP and TBP–MF) was evaluated using a thermogravimetric analyzer (TGA–1000 °C, Shanghai Innuo Precision Instruments Co., Ltd., Shanghai, China) and a differential scanning calorimeter (DSC8500, PerkinElmer, Waltham, MA, USA) [23]. For TGA analysis, approximately 10 mg of lyophilized sample was placed in a crucible and heated from 30 °C to 500 °C at a constant heating rate of 10 °C/min under a nitrogen atmosphere. The weight-loss curves of the samples as a function of temperature were recorded. For DSC analysis, approximately 10 mg of sample was sealed in an aluminum crucible and scanned from 25 °C to 150 °C at a heating rate of 10 °C/min under nitrogen, using an empty aluminum crucible as a reference. The thermal stability and thermal transition characteristics of the peptides were comprehensively evaluated by analyzing the mass loss and endothermic/exothermic features [24]. Thermal stability analysis (TGA/DSC) was performed to compare the thermal behavior of TBP and TBP–MF as an indicator of their physicochemical robustness and structural organization after hydrolysis and gel-filtration fractionation. These measurements help distinguish differences in composition/ordering between the crude hydrolysate and the enriched fraction, and provide basic stability information relevant to potential downstream processing and formulation (e.g., functional foods or pharmaceutical preparations).

### 2.5. Fourier Transform Infrared Spectroscopy (FTIR)

The secondary structures of TBP and TBP–MF were determined using a Fourier transform infrared spectrometer (Thermo Nicolet iS 10, Thermo Fisher Scientific, Waltham, MA, USA). Briefly, 2 mg of lyophilized peptide powder was thoroughly mixed with spectroscopic-grade KBr and pressed into pellets for measurement [25]. The scanning range was set from 4000 to 400 cm^−1^ with a resolution of 4.00 cm^−1^, and 32 scans were accumulated for each sample. The resulting spectra were baseline-corrected and normalized to analyze the absorption characteristics of the amide I and amide II bands.

To obtain quantitative information on the secondary structures, the amide I region (1600–1700 cm^−1^) was imported into PeakFit (version 4.12) for peak deconvolution and Gaussian curve fitting [26]. Based on the secondary-structure assignments corresponding to specific peak positions, the proportion of each sub-peak area relative to the total area of the amide I band was calculated to characterize the relative content of each secondary structure in the samples.

### 2.6. Circular Dichroism (CD) Spectra

The circular dichroism spectra of the target protein peptides were recorded using a CD spectrometer (Applied Photophysics Ltd., Leatherhead, UK). During the test, peptide samples were dissolved in deionized water to a concentration of 1 mg/mL and filtered through a 0.22 μm microporous membrane. Scanning was performed at room temperature within the wavelength range of 190–260 nm. The resulting spectra were subjected to background subtraction and smoothing to characterize the secondary-structure composition of the peptides in solution [27]. CD spectroscopy was used as a comparative tool to assess ensemble-averaged conformational features of TBP and TBP-MF in aqueous solution, complementary to the solid-state information obtained by FTIR.

### 2.7. Micromorphology and Particle Size Analysis

The micromorphology and particle size distribution of the peptides were characterized using a field-emission scanning electron microscope (FE–SEM; SU5000, Hitachi High–Tech Corporation, Tokyo, Japan) and a laser particle size analyzer (Malvern Panalytical, Malvern, UK), respectively. For SEM analysis, the lyophilized peptide powder was uniformly spread on conductive adhesive and sputter-coated with metal under vacuum. Images were captured at an accelerating voltage of 5.0 kV and at various magnifications to observe surface morphology [24]. For ImageJ (version 2.0, National Institutes of Health, Bethesda, MD, USA)-based projected-area analysis, domains with elongated, lamellar, fractured, or sheet-like outlines were classified as flake-like features, whereas compact domains with approximately circular outlines were classified as spheroidal features; the classification was used as a comparative morphological descriptor.

For particle size analysis, peptide samples were dissolved in PBS at 1 mg/mL. Particle size was measured by dynamic light scattering (DLS). Samples were transferred to disposable polystyrene cuvettes and equilibrated at 25 °C for 2 min before measurement. The Z-average and polydispersity index (PDI) were recorded. The ζ-potential was determined by electrophoretic light scattering (ELS) using a disposable folded capillary electrophoresis cell (DTS1070; Malvern Panalytical, Malvern, UK). The ζ-potential was converted from the electrophoretic mobility using the Smoluchowski model. All measurements were performed in triplicate, and the average values were reported [28].

### 2.8. Molecular Mass Distribution by MALDI–TOF Mass Spectrometry

The molecular mass of the samples was determined using a matrix-assisted laser desorption/ionization time-of-flight mass spectrometer (MALDI–TOF MS; MALDI–8030, Shimadzu, Kyoto, Japan). Samples were dissolved in deionized water and appropriately diluted. α–cyano–4–hydroxycinnamic acid (CHCA) was used as the matrix. The sample and matrix solution were mixed at a 1:1 volume ratio and spotted onto a MALDI target plate, which was then air-dried at room temperature to form co-crystals. Mass signals were acquired in positive-ion reflectron mode over a mass-to-charge ratio (*m*/*z*) range of 200–5000. External mass calibration was performed using peptide standards. The obtained spectra were subjected to baseline subtraction and smoothing. The molecular masses were calculated based on the *m*/*z* values of the prominent peaks to analyze the molecular mass distribution of the samples [20].

### 2.9. Peptide Sequence Identification by Q–TOF MS/MS

TBP and TBP–MF samples were dissolved in ultrapure water to 1 mg/mL and centrifuged at 12,000 rpm for 10 min. The supernatant was filtered through a 0.22 μm microporous membrane and placed in a liquid chromatography sample vial. An injection volume of 1 μL was used for analysis on a UHPLC–Q–TOF–MS/MS system (Shimadzu LCMS–9030, Shimadzu, Kyoto, Japan). Data were processed using LabSolutions Insight (version 3.8, Shimadzu Corporation, Kyoto, Japan). Peptide sequences were identified using a database search method, where the acquired tandem mass spectra (MS/MS) were compared against the tuna protein database (UniProt/NCBI). Amino acid sequences were screened and determined based on the accurate mass matching of precursor and fragment ions [19,29].

### 2.10. Potential Bioactivity Screening Based on BIOPEP–UWM

The potential biological activities of the peptide sequences were screened in silico using the BIOPEP–UWM online database (https://biochemia.uwm.edu.pl/biopep–uwm/, accessed on 4 November 2025). The candidate peptide sequences were submitted to the “Profiles of potential biological activity” module of the database to generate analysis reports and record matching results with known bioactive fragments. Particular attention was paid to activity tags related to neuromodulation and angiotensin-converting enzyme (ACE) inhibition to evaluate the peptides’ potential functional activities. Concurrently, PeptideRanker (http://distilldeep.ucd.ie/PeptideRanker/, accessed on 5 November 2025) was employed to predict and score the bioactivity of TBP and TBP–MF [30,31].

### 2.11. CCK-8-Based Cytocompatibility Assay

Highly differentiated rat pheochromocytoma (PC12) cells (Shanghai Fuheng Biotechnology Co., Ltd., Shanghai, China) were seeded in T25 culture flasks and maintained in complete medium (10% fetal bovine serum and 1% penicillin–streptomycin solution) at 37 °C in a 5% CO_2_ incubator. When the cell density reached approximately 80% confluence, the cells were seeded into 96-well plates at 1 × 10^4^ cells/well. The cells were then co-incubated with various concentrations of target protein peptides (0.01, 0.05, 0.1, 0.5, 1, and 5 mg/mL) for 24 h, while the control group received an equal volume of complete medium. Following incubation, 100 μL of medium containing 10% Cell Counting Kit–8 (CCK–8; M4839, AbMole, Houston, TX, USA) was added, and the plates were incubated for an additional 30 min in the dark, and the absorbance was measured at 450 nm [32]. The CCK–8 assay uses WST–8, a highly water-soluble tetrazolium salt, which is reduced by cellular dehydrogenases in metabolically active cells to generate a water-soluble formazan dye. Therefore, the OD_450_ signal reflects cellular metabolic activity and is commonly used as an indirect indicator of viable cell number and proliferative activity under growth conditions.

### 2.12. Quantitative Determination of Related Factor Levels

The levels of sleep-regulating factors (BH4, 5–HT, and GABA) were quantified by ELISA in highly differentiated PC12 cells after TBP or TBP–MF treatment, and the pathway-critical enzymes TPH2 and GCH1 were measured in parallel. Cells were treated with TBP or TBP–MF at 0.01, 0.05, 0.1, 0.5, 1, and 5 mg/mL for 24 h, while the control group received an equal volume of medium. After the 24 h treatment, cells were harvested, washed with PBS, and lysed. The supernatant was centrifuged and collected as the test sample. To minimize the impact of cell number variations, total protein quantification was performed on samples to normalize results. Each indicator was detected using the corresponding commercial ELISA kits (Jiangsu Meimian Industry Co., Ltd., Yancheng, China). Standard curve preparation, sample addition, incubation, washing, color development, and reaction termination were performed as instructed. Absorbance was measured at 450 nm. Target factor concentrations in samples were calculated from the standard curve and expressed as either concentration or content normalized to total protein [32].

### 2.13. Statistical Analysis

All experiments were performed in triplicate, and data are presented as the mean ± standard deviation (SD). Statistical analyses and figure generation were performed using GraphPad Prism (v10.1). Statistical results were independently verified using SPSS (IBM SPSS Statistics 27). Between-group comparisons were conducted using one-way or two-way analysis of variance (ANOVA), as appropriate. *p* < 0.05 was considered statistically significant.

## 3. Results and Discussion

### 3.1. Sephadex G-25 Gel Filtration Chromatography

**Figure 1:**
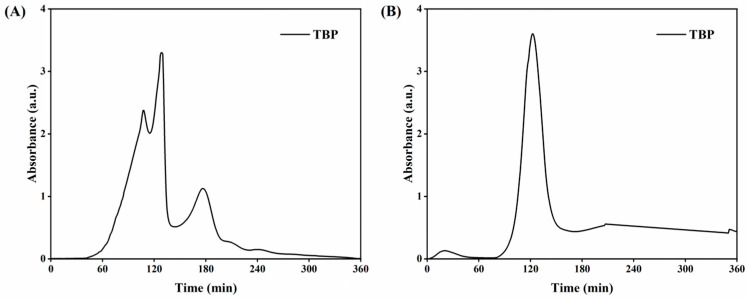
Sephadex G-25 gel filtration chromatograms of the peptide fractions. (**A**) Elution profile of TBP (the <3 kDa ultrafiltrate fraction); (**B**) TBP–MF corresponds to the dominant, well-resolved peak (tubes 11–13) collected from the Sephadex G-25 chromatogram.

The crude hydrolysate was first subjected to ultrafiltration (MWCO 3 kDa) to obtain the <3 kDa peptide fraction (TBP). To further reduce compositional heterogeneity and enrich the predominant components, TBP was fractionated by Sephadex G-25 gel filtration. As shown in the chromatograms (Figure 1A), TBP exhibited multiple elution peaks, indicating that the <3 kDa ultrafiltrate still contained several peptide populations differing in size or composition. Integration of the peak areas revealed that TBP resolved into three major peaks, accounting for 39.1%, 33.4%, and 27.5% of the total area, respectively, confirming its heterogeneous nature.

The dominant and well-resolved chromatographic peak of TBP (eluting around 120 min, tubes 11–13) was collected and referred to as TBP–MF. This peak was selected because it showed the highest A220 signal and good separation from neighboring peaks, enabling the collection of a relatively compositionally enriched fraction for subsequent characterization and bioactivity evaluation. Other elution peaks were also considered. The late elution signal around 180 min was examined using a BCA assay and showed no detectable protein/peptide content, suggesting that this peak mainly reflected non-peptidic components. In addition, earlier peaks correspond to higher-molecular-mass species (consistent with the size-exclusion elution order, where larger molecules elute first) and were therefore not aligned with our objective of obtaining a low-molecular-mass peptide fraction. For these reasons, we selected the dominant, well-resolved peak at 120 min (tubes 11–13) as the enriched peptide fraction (TBP–MF) for downstream characterization and cellular evaluation. In contrast to TBP, TBP–MF displayed a dominant chromatographic peak with markedly reduced signals from minor components, suggesting that gel filtration effectively enriched the target fraction and improved sample homogeneity. Collectively, Sephadex G-25 fractionation allowed us to isolate a compositionally enriched peptide fraction for downstream structural characterization and cellular evaluation, while other elution regions may also warrant future investigation.

### 3.2. Thermal Stability Analysis

**Figure 2:**
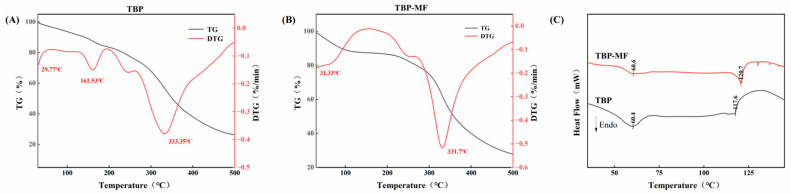
TGA/DTG thermogravimetric curves of TBP (**A**) and TBP–MF (**B**). DSC curves of TBP and TBP–MF (**C**).

The thermal stability and phase transition behaviors of TBP and TBP–MF were evaluated using TGA/DTG and DSC (Figure 2). TBP exhibited a multi-stage weight-loss profile during heating (Figure 2A). The derivative thermogravimetry (DTG) curve highlighted three distinct degradation events: moisture evaporation at 29.77 °C, a secondary degradation peak at 161.53 °C, and a primary thermal decomposition peak at 333.35 °C. The peak near 160 °C likely corresponds to the breakdown of thermally unstable small molecules or impurities present in the crude mixture.

In contrast, the major TBP–MF fraction displayed a significantly more homogeneous degradation profile (Figure 2B). The secondary degradation peak in the 160–168 °C range was markedly attenuated, and the primary decomposition occurred at 331.7 °C. Comparable multi-stage degradation behaviors have been reported for protein/peptide-based materials, where low-temperature events are often associated with moisture/volatiles, and higher-temperature steps reflect backbone/aggregate breakdown [33]. Quantitatively, the temperatures corresponding to 20% and 50% weight loss for TBP–MF were 269 °C and 359 °C, respectively, which were notably higher than those of TBP (233 °C and 351 °C). Furthermore, TBP–MF retained a higher residual mass at 500 °C. This enhancement in thermal stability suggests that the G-25 fractionation effectively removed labile components and facilitated the formation of a more compact molecular arrangement.

DSC analysis (Figure 2C) corroborated these findings. Both samples exhibited endothermic peaks near 60 °C (likely solvent desorption) and a major thermal transition between 118 and 121 °C, with TBP–MF showing a slightly higher peak temperature, suggesting a higher energetic barrier for conformational/interaction rearrangements. Such DSC shifts are often observed when peptide ensembles become more uniform and more strongly associated (e.g., via tighter hydrogen bonding or aggregate packing), which typically raises transition temperatures relative to heterogeneous mixtures [34].

### 3.3. Secondary-Structure Fingerprints

FTIR analysis was used here not simply to confirm the expected presence of amide bands in peptide-containing samples, but to compare spectral differences between TBP and TBP-MF. The characteristic band assignments are listed in Table 1. The amide I maximum shifted slightly from 1654.1 cm^−1^ in TBP to 1652.3 cm^−1^ in TBP-MF, and TBP-MF also exhibited a more distinct amide II band at 1550.3 cm^−1^. These spectral features suggest differences in the hydrogen-bonding environment and reduced conformational heterogeneity after Sephadex G-25 fractionation. Thus, the FTIR data are interpreted here as comparative indicators of ensemble-level structural organization in the two peptide mixtures, rather than as generic evidence of peptide character [35].

**Figure 3:**
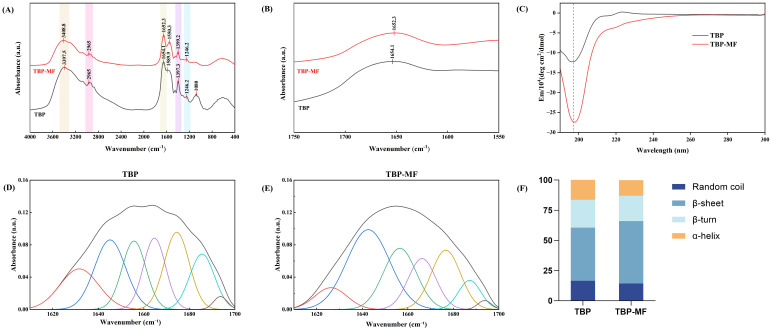
FTIR spectra of TBP and TBP–MF are shown in (**A**), with the corresponding enlarged view in (**B**), and the circular dichroism spectra are presented in (**C**). Gaussian multi-peak fitting of the amide I region was performed using PeakFit software to obtain the deconvoluted curves for TBP in (**D**) and for TBP–MF in (**E**), where the black curve represents the experimental spectrum and the colored curves represent the individual fitted component peaks.. The bar chart (**F**) summarizes the relative secondary-structure contents calculated from the integrated areas of the resolved amide I sub-bands.

To better resolve structural information in the amide I region, Gaussian peak fitting was carried out, and the area contribution of each resolved band was quantified. As shown in Figure 3F, TBP was mainly composed of beta-sheet structures, accounting for 43.99%, together with 16.41% alpha-helix, 23.03% beta-turn, and 16.57% random coil. In contrast, TBP–MF showed a further increase in the beta-sheet fraction to 51.70%, while alpha-helix decreased to 13.03%, beta-turn decreased to 20.89%, and random coil decreased to 14.39%. This shift suggests that TBP-MF exhibited a greater contribution from β-sheet-related spectral components at the mixture level, consistent with a more ordered hydrogen-bonding environment than the crude TBP fraction [36]. Figure 3F summarizes the results of Gaussian deconvolution of the measured spectra performed under identical fitting settings for all samples. Because TBP and TBP–MF are peptide mixtures, the deconvoluted peak areas represent ensemble-averaged spectral contributions rather than secondary-structure assignments for individual peptides.

**Table 1:** FTIR band assignments of TBP and TBP–MF.

Band	Frequencies of Amide Bands (cm^–1^)		Assignment
	TBP	TBP–MF	
Amide A	3397.5	3408.8	N–H stretching (overlap with O–H)
Amide B	2965.0	2965.0	Asymmetric C–H stretching
Amide I	1654.1	1652.3	C=O stretching
Amide II	1589.9	1550.3	N–H bending coupled with C–N stretching
Amide III	1246.2	1246.2	

CD spectroscopy was used as a comparative tool to assess the ensemble-averaged secondary-structure signatures of TBP and TBP–MF in aqueous solution, thereby complementing the FTIR analysis. Because both samples are peptide mixtures rather than single defined sequences, the CD data were interpreted qualitatively, with emphasis on relative spectral differences rather than absolute secondary-structure percentages. Both TBP and TBP–MF exhibited a strong negative band near 200 nm and lacked the characteristic double minima at 208 and 222 nm typical of α-helical structures, indicating that both samples were predominantly disordered/random-coil-like in solution. Notably, TBP–MF showed a more pronounced negative ellipticity near 200 nm, suggesting a relative conformational shift compared with TBP, rather than direct evidence of β-sheet enrichment in solution. The apparent difference between the FTIR and CD results is not unexpected and can be attributed to the distinct sample states probed by the two techniques. FTIR primarily reflects structural organization in the lyophilized or solid-state sample and is particularly sensitive to hydrogen-bond reorganization and intermolecular packing, whereas CD captures the ensemble-average conformations of flexible peptide mixtures in solution. Lyophilization may therefore enhance solid-state β-sheet-related organization that is not equivalently represented in the solution-state CD spectra. Taken together, these results highlight the value of combining FTIR and CD to characterize peptide structural behavior across different physical states, rather than relying on either method alone [37].

### 3.4. Microstructure (SEM) and Colloidal Behavior (DLS/ζ-Potential)

**Figure 4:**
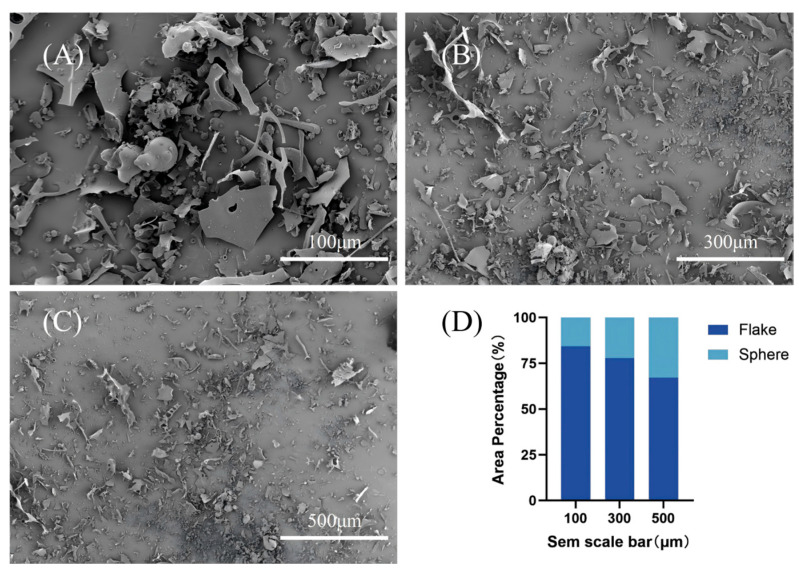
SEM micrographs of TBP at different scales: (**A**) 100 μm, (**B**) 300 μm, and (**C**) 500 μm. (**D**) Stacked bar chart showing the relative area fractions of flake-like and spherical features in TBP across the three scales, quantified by projected-area analysis using ImageJ.

**Figure 5:**
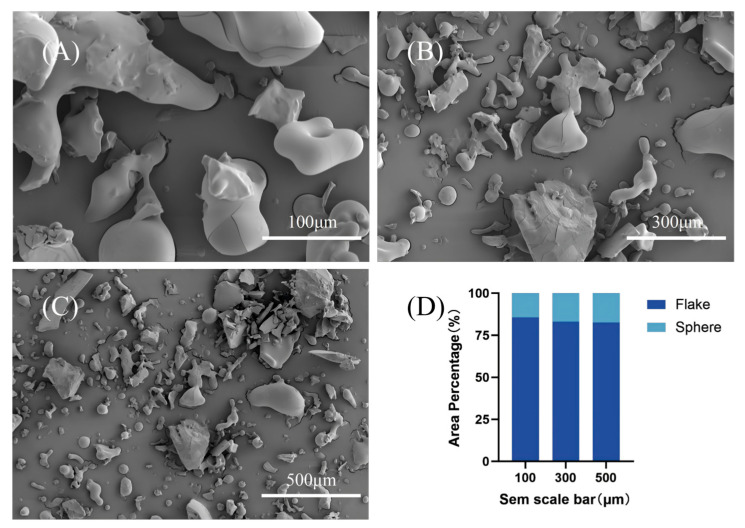
SEM micrographs of TBP–MF at different scales: (**A**) 100 μm, (**B**) 300 μm, and (**C**) 500 μm. (**D**) Stacked bar chart showing the relative area fractions of flake-like and spherical features in TBP–MF across the three scales, quantified by projected-area analysis using ImageJ.

To complement the molecular-level analyses, SEM and DLS/ζ-potential measurements were used here as comparative physicochemical tools to characterize fraction-level differences in morphology, dispersion behavior, and association tendency between TBP and TBP-MF. Before imaging, samples were sputter-coated with a thin conductive metal layer to mitigate surface charging [38]. The surface microstructures—including particle architecture, roughness, pore features, and aggregation patterns—were then examined by SEM. SEM micrographs of TBP (Figure 4) and the corresponding fraction TBP–MF (Figure 5) were obtained with scale bars of 100, 300, and 500 μm. Clear morphological differences were observed between the two peptide preparations; however, both materials were composed mainly of irregular, lamellar/flake-like fragments. Notably, TBP—collected as a heterogeneous mixture after ultrafiltration—showed a strongly non-uniform distribution, characterized by irregular blocky agglomerates together with relatively large fractured pieces. ImageJ-based area measurements across multiple fields of view revealed that flake-like domains accounted for 84.23%, 77.76%, and 67.16% of the projected area at 100, 300, and 500 μm, respectively [39]. In contrast, the proportion of spheroidal features increased as the observation window expanded, rising from 15.77% to 32.84%, suggesting that small spherical particles are captured more frequently when larger fields of view are analyzed.

Following purification through a dextran gel column, the morphology of TBP–MF underwent significant alteration. Although a small number of irregular particles remained observable under SEM, the overall structure predominantly exhibited a lamellar arrangement, accompanied by numerous elongated fibrous aggregates. Quantification using ImageJ revealed that sheet-like structures constituted the majority of the projected area (82.64–85.63%) across scale bars of 100, 300, and 500 μm, whereas spherical features accounted for only 14.37–17.36%. This suggests a greater degree of morphological consistency across the observed fields. Under the present preparation and imaging conditions, the enriched TBP-MF fraction displayed a more uniform lamellar/fibrous morphology than the crude TBP mixture. This observation is interpreted here as a comparative fraction-level difference rather than as direct evidence of a distinct self-assembly mechanism. This behavior is consistent with the established literature on freeze-drying, where dehydration promotes intermolecular contacts and structural reorganization (often favoring β-sheet formation), leading to the architectures observed here [40].

**Figure 6:**
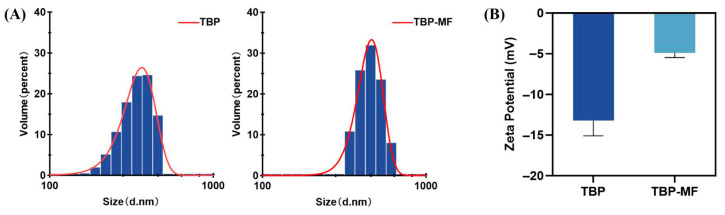
(**A**) Particle size distribution and (**B**) zeta potential of TBP and TBP–MF.

Dynamic light scattering (DLS) and ζ-potential measurements were used here to compare the colloidal behavior of TBP and TBP-MF as peptide mixtures under identical re-dispersion conditions. These results therefore describe fraction-level dispersion and association behavior of the tested preparations, rather than intrinsic physicochemical properties of individual peptide molecules. DLS reflects particle diffusion behavior and hydrodynamic diameter in solution, which are significantly influenced by aggregation/shape. In our case, Malvern measurements (Figure 6A,B) showed that both TBP and TBP–MF formed submicron colloidal assemblies in water, comparable to peptide-/protein-based delivery systems reported in the literature—for example, nanoliposomes encapsulating fish gelatin peptide fractions exhibited particle sizes spanning 134–621 nm, with composition-dependent shifts [41]. Likewise, chitosan-coated nanoliposomes carrying rainbow trout skin-derived antioxidant peptides typically displayed mean sizes of 163–234 nm, demonstrating that peptide–carrier or peptide–peptide interactions can readily tune the DLS diameter into the submicron regime [42] Consistent with these precedents, TBP showed a broader, flatter volume-weighted distribution, with a main peak region around 295–396 nm, whereas TBP–MF exhibited a more concentrated distribution with nearly all volume contribution within 342–615 nm and a dominant mode at 459 nm. The cumulative percentiles further yielded D10/D50/D90 values of 227/321/416 nm for TBP and 339/422/525 nm for TBP–MF, indicating that TBP–MF more readily forms larger hydrated associates in solution.

The ζ-potential results (Figure 6B) corroborate the higher tendency toward aggregation observed for TBP–MF. While TBP exhibited a potential of −13.23 ± 1.86 mV, TBP–MF showed a significantly lower magnitude of −4.91 ± 0.57 mV. Notably, the very low absolute zeta potential of TBP–MF indicates weak electrostatic repulsion and poor colloidal stability, consistent with an increased tendency toward aggregation or re-association in suspension. A similar scenario of reduced electrostatic stabilization has been documented in peptide-loaded nanoliposome systems, where ζ-potential values near 0–9 mV were associated with particle instability and size growth [41]. Importantly, our FTIR findings—which reveal that TBP–MF is more ordered and dominated by β-sheets—provide a structural rationale for the trends observed in DLS and ζ-potential analyses. In biopharmaceutical proteins, the conversion to intermolecular β-sheets is frequently associated with particle size increases, suggesting a possible link between secondary-structure reorganization and physical aggregation [43]. Finally, the observation that processing history influences this state is supported by the formulation literature, which indicates that lyophilization can amplify ordered association. The dehydration process often increases β-sheet content while reducing α-helical content, predisposing susceptible fractions (such as TBP–MF) to stronger association upon reconstitution [44]. From an application perspective, these physicochemical differences may be relevant to handling, dispersion stability, formulation, and the effective exposure of peptide fractions in aqueous or cell-based systems. However, these morphology- and colloid-related measurements should be interpreted as supportive context rather than as direct explanations of the observed neurochemical responses.

### 3.5. Mass Spectral Analysis

**Figure 7:**
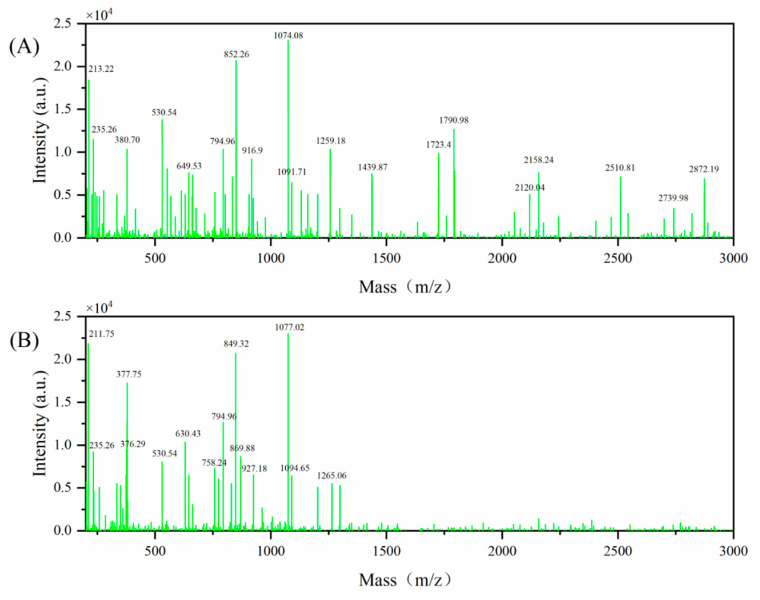
Mass spectra of TBP (**A**) and TBP–MF (**B**) collected over 200–3000 *m*/*z*.

MALDI–TOF analysis (200–3000 *m*/*z*) showed that TBP contained peptide signals overlapping with those observed in TBP–MF, together with additional higher-mass components extending to approximately 3000 *m*/*z*. By contrast, the post-chromatography TBP–MF fraction was mainly distributed within the lower range of approximately 200–1265 *m*/*z*. Thus, the principal result in Figure 7 is that Sephadex G-25 fractionation enriched a lower-mass subpopulation from the broader TBP peptide mixture. These findings indicate that Sephadex G-25 fractionation yielded a TBP-MF fraction with a narrower molecular-mass distribution. The higher-mass peptide populations present in TBP are therefore interpreted not as contaminants, but as non-enriched components outside the selected fraction studied here.

### 3.6. Peptide Activity Prediction

Using UHPLC–Q–TOF–MS/MS, the peptide profiles of TBP and the Sephadex G-25-enriched fraction (TBP-MF) were analyzed. To focus on key contributors to bioactivity, the 15 most abundant peptides with the highest identification confidence were selected from each fraction for detailed characterization (Table 2). Within our dataset, both groups contained several relatively higher-scoring candidate peptides, but the score distribution of TBP–MF showed an overall upward shift compared with TBP. Importantly, none of the identified peptides exceeded the commonly used PeptideRanker threshold of 0.5. Therefore, the PeptideRanker results are interpreted here as a tool for relative prioritization within this dataset rather than as direct evidence of strong bioactivity.

We first used PeptideRanker to prioritize peptides identified in TBP and TBP–MF. This tool was developed to estimate the likelihood that a peptide is bioactive across diverse functional classes, and it has been widely adopted as a practical front-end filter for complex peptidomics datasets before targeted validation [45]. In our dataset, both groups contained multiple relatively higher-scoring candidates, yet the TBP–MF set exhibited a consistent shift toward higher scores. The top peptide in TBP–MF was DLATNPKPR (0.4303), followed by LSGQDLR (0.4033), SGFQAEYVR (0.3804), and HMADYELR (0.3761), whereas the highest-ranked TBP peptides were VVGLPGTR (0.3030) and MSTNPKPQR (0.2913). Because PeptideRanker is intended for relative prioritization rather than target-specific potency prediction, we interpret this upward shift as evidence that Sephadex G-25 fractionation enriched a peptide subset with higher overall bioactivity propensity, consistent with prior in silico-guided discovery workflows that combine ranking with functional annotation to triage candidates for downstream assays [46].

To contextualize the predicted functional space, we next mapped both peptide sets using BIOPEP–UWM, a curated resource commonly used to identify known bioactive fragments embedded within peptide sequences and to interpret peptidomics results [47]. Fragmentomics indicated that ACE-inhibitory and DPP–IV-inhibitory motifs were abundant in both TBP and TBP–MF, which is consistent with the frequent appearance of these two activity classes in food-protein peptide libraries [48]. This pattern is also consistent with established structure–activity relationships for ACE-inhibitory peptides, in which hydrophobic and/or aromatic residues often contribute favorably to ACE binding. Therefore, the presence of multiple ACE-related fragments in our datasets provides mechanistic support for the predicted annotations rather than representing a purely database-derived assignment [49]. In addition to these shared motif classes, TBP–MF showed a relative enrichment of fragments annotated as neuroactive or neuroprotective. Although ACE- and DPP-IV-related motifs remained the predominant predicted activity classes in both groups, this observation suggests that fractionation may have shifted the annotation profile toward more neuro-related features while also increasing the overall PeptideRanker scores. This tendency is broadly compatible with recent studies on sleep-related food-derived peptides, which suggest that short peptides may influence sleep through neurotransmitter-associated pathways, including GABAergic regulation and other neuromodulatory mechanisms [50]. Similarly, studies on casein hydrolysates have reported that short peptides containing aromatic residues are enriched among sleep-enhancing candidates, supporting the possibility that certain TBP–MF peptides may have a greater likelihood of interacting with neuro-related targets [51]. Because ACE also participates in the brain renin–angiotensin system, ACE-related motifs may carry indirect neurobiological relevance; however, this possibility was not experimentally validated in the present study.

At the sequence level, TBP–MF included hydrophobic motifs such as GILFV (from GILFVGSSR) and YF (from VTVPAYFNK), and it contained multiple aromatic residues (Phe, Tyr, Trp). These features offer a molecular explanation for the secondary-structure trends observed in our FTIR/CD analyses, because hydrophobic packing and aromatic interactions are known drivers of peptide association and higher-order organization. Finally, while blood–brain barrier (BBB) transport must be experimentally confirmed, prior work has emphasized that hydrophobicity can enhance BBB permeability or support BBB-shuttle strategies for peptide delivery, providing a reasonable basis for proposing that TBP–MF may possess improved CNS-oriented potential compared with the crude TBP mixture.

Taken together, the higher PeptideRanker distribution in TBP–MF, the dense ACE/DPP–IV fragment signature shared by both groups, and the additional neuroactive motif enrichment in TBP–MF collectively support our experimental strategy: membrane-based fractionation enriches a peptide subset with higher predicted bioactivity and a functional annotation profile that is consistent with subsequent neuro-related assays.

### 3.7. CCK-8-Based Cytocompatibility and Functional Screening in Differentiated PC12 Cells

**Figure 8:**
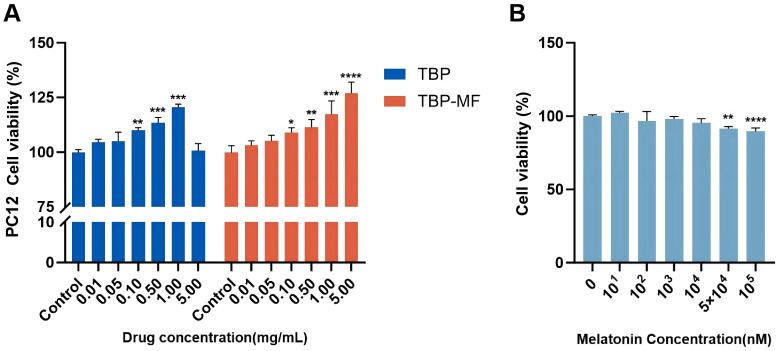
(**A**) CCK-8 readout of differentiated PC12 cells cultured with different concentrations of TBP and TBP-MF for 24 h. (**B**) CCK-8 response profile of melatonin under the same assay conditions. * *p* < 0.05, ** *p* < 0.01, *** *p* < 0.001, and **** *p* < 0.0001 compared with the control group. Data are presented as mean ± SD (*n* = 3).

Highly differentiated PC12 cells were used as a neuron-like model, a strategy commonly adopted for neuronal screening [22]. The CCK-8 assay was used here as an initial cytocompatibility and functional-screening step to identify concentration ranges that did not reduce cellular metabolic activity and that provided measurable cellular responses before the downstream neurochemical analyses (Figure 8A). Within 0.01–5.00 mg/mL, neither TBP nor TBP-MF reduced the CCK-8 readout relative to the control; instead, both preparations produced concentration-dependent increases in the OD_450_ signal under the present conditions. This trend is consistent with reports that peptide mixtures/hydrolysates can improve PC12 survival under stressful conditions or in functional-screening settings, such as oat-protein-derived peptides that show protective effects in PC12 oxidative-stress models [52]. A clear difference emerged in the dose–response shape between TBP and TBP–MF. TBP reached its maximal increase in CCK–8 signal at 1.00 mg/mL and then declined toward the control level at 5.00 mg/mL, suggesting a “moderate–dose optimum”. By contrast, TBP–MF showed a more monotonic increase, peaking at 5.00 mg/mL. This divergence is mechanistically plausible because peptide systems frequently display concentration-dependent behavior in cell assays: as concentration increases, peptide–peptide interactions and micro-aggregation can change the effective bioavailable fraction and thereby flatten or reverse an otherwise positive response—whereas a more compositionally uniform fraction can maintain a stronger effect at higher loading [53]. Therefore, TBP may approach functional saturation or exhibit reduced effective availability at higher concentrations, whereas TBP–MF retains a larger enhancement window, consistent with its greater compositional homogeneity after fractionation.

The CCK-8 response profile of the comparative positive reference compound melatonin is shown in Figure 8B. At lower to intermediate concentrations, the CCK-8 readout remained broadly comparable to the control, whereas the highest tested concentrations showed a limited decrease in signal. This indicates concentration-dependent responsiveness under the present assay conditions, but should not be overinterpreted as evidence of strong cytotoxicity.

**Figure 9:**
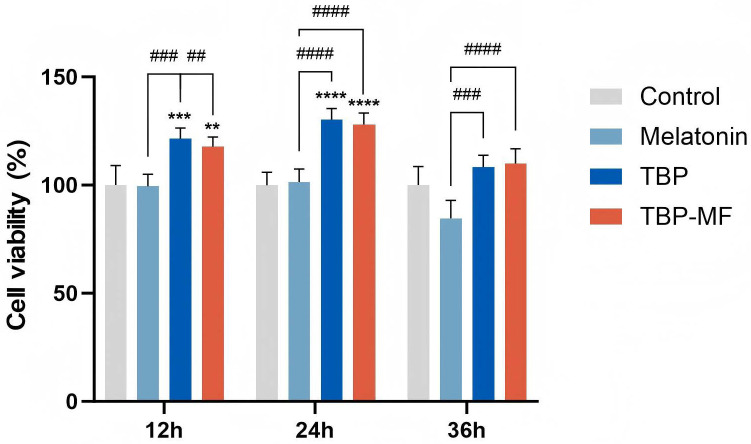
Time-dependent effects of TBP and TBP–MF at their optimal doses on PC12 cells. ** *p* < 0.01, *** *p* < 0.001, and **** *p* < 0.0001 compared with the control group. ^##^ *p* < 0.01, ^###^ *p* < 0.001, ^####^ *p* < 0.0001 indicated statistical significance compared with the positive control melatonin. Data are presented as mean ± SD (*n* = 3).

Based on the CCK-8 screening, TBP (1.00 mg/mL) and TBP-MF (5.00 mg/mL)—the concentrations giving the strongest increase in the CCK-8 readout for each fraction—were selected for the time-course experiment shown in Figure 9.

Both peptide preparations increased PC12 viability at as early as 12 h, reached a maximum at 24 h, and then showed a modest attenuation by 36 h, while remaining slightly above the control level. This temporal profile suggests that TBP and TBP–MF primarily improve the early metabolic/functional state and stress tolerance of PC12 cells, rather than driving a continuous, linear increase over time—an interpretation consistent with PC12 studies where cellular responses often peak around 24 h and then plateau or partially decline as the system adapts [54]. Moreover, food-derived peptide studies in PC12 commonly assess protection at ~24 h and report viability rescue linked to stress-response signaling (e.g., PI3K/AKT or Nrf2-related pathways), supporting the idea that the major benefit can manifest within the first day and may not keep rising indefinitely with prolonged incubation [55].

### 3.8. Effects of Tuna By-Product Peptides on the Upregulation of TPH2 and GCH1 and the Synthesis of Sleep Factors BH4, GABA, and 5–HT

**Figure 10:**
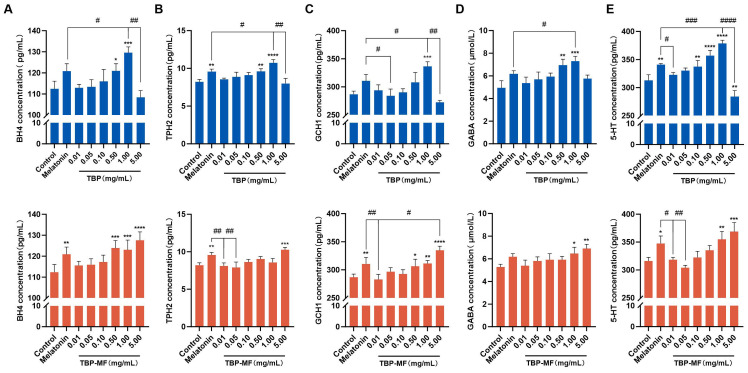
Effects of TBP and TBP–MF (0.01–5 mg/mL, 24 h) on intracellular (**E**) 5–HT, (**A**) BH4, and (**D**) GABA levels, as well as (**B**) TPH2 and (**C**) GCH1 expression in PC12 cells. * *p* < 0.05, ** *p* < 0.01, *** *p* < 0.001, and **** *p* < 0.0001 compared with the control group. ^#^ *p* < 0.05, ^##^ *p* < 0.01, ^###^ *p* < 0.001, ^####^ *p* < 0.0001 indicate statistical significance compared with the positive control melatonin. Data are presented as mean ± SD (*n* = 3).

The ELISA measurements shown in Figure 10 were conducted across the same full concentration range used in the initial screening (0.01, 0.05, 0.1, 0.5, 1.0, and 5.0 mg/mL). Because TBP and TBP–MF exhibited distinct dose–response patterns in the CCK–8 assay, the neurotransmitter-related responses were interpreted separately for each fraction. ELISA analysis showed that both TBP and TBP–MF increased intracellular 5–HT and GABA after 24 h of treatment across the tested concentration range, and these increases were accompanied by parallel elevations in BH4, GCH1, and TPH2, indicating coordinated modulation of BH4, GCH1, TPH2, and neurotransmitter levels under the present experimental conditions. BH4 is a required cofactor for aromatic amino acid hydroxylases, including tryptophan hydroxylase (TPH) in the serotonin biosynthetic pathway, and intracellular BH4 availability is largely governed by the activity of GCH1 [56]. As the first and rate-limiting enzyme in de novo BH4 biosynthesis, GCH1 upregulation is expected to promote BH4 accumulation [57]. In neurons, TPH2 is the key isoform controlling brain 5–HT synthesis; genetic perturbation of TPH2 reduces serotonin production and can be detected even in PC12-based systems, supporting the biological relevance of the BH4–TPH2–5–HT pathway-associated change in this model [58]. Within the TBP group, BH4, GCH1, and TPH2 were all significantly increased versus the control, while 5–HT peaked at 1.0 mg/mL and declined at 5.0 mg/mL, showing a typical “moderate–dose optimum” profile. Such bell-shaped responses are frequently attributed to pathway saturation or negative-feedback constraints; for serotonin-related pharmacology, one mechanistic example is that 5–HT_1A autoreceptor-mediated feedback can reduce efficacy at higher exposure, producing a bell-shaped dose–response curve [59]. More broadly, biphasic (stimulatory-then-attenuated) responses are widely observed in biological systems and are often discussed under the framework of hormesis, where increasing dose can shift the balance from adaptive activation to counter-regulation. By contrast, TBP–MF displayed a smoother concentration dependence: BH4, GCH1, TPH2, and 5–HT rose progressively and reached maxima at 5.0 mg/mL. Taken together, these results suggest that TBP–MF may be less constrained by high-dose feedback or bioavailability loss, whereas TBP—being more heterogeneous—could be more sensitive to homeostatic regulation or concentration-dependent physicochemical changes. However, the present results remain correlational and do not establish a causal hierarchy among GCH1, BH4, TPH2, and 5–HT.

GABA levels were significantly increased in both TBP- and TBP–MF-treated cells. This direction is consistent with the known involvement of GABAergic signaling in sleep-related neurophysiology. However, in the present study, the observed increase in intracellular GABA should be interpreted as a neurochemical change associated with sleep-related pathways rather than as direct evidence of sleep-promoting efficacy. Previous studies on sleep-related food-derived peptides have also reported concurrent changes in GABA and 5-HT as part of broader neurochemical readouts, which are broadly consistent with the neurotransmitter shifts observed here [60,61]. More direct mechanistic validation, including inhibitor-based experiments, will be necessary to determine whether the GABA-related changes observed in this study play a causal role in any sleep-related functional effect.

## 4. Conclusions

In this study, low-molecular-mass peptides derived from yellowfin tuna processing by-products were structurally refined by Sephadex G–25 fractionation. The major fraction (TBP–MF) exhibited improved compositional homogeneity, enhanced β-sheet enrichment, increased thermal stability, and more ordered self-association behavior compared with the crude hydrolysate. In differentiated PC12 cells, TBP–MF demonstrated stable, concentration-dependent enhancement of cellular activity and significantly increased intracellular levels of BH4, serotonin, and γ-aminobutyric acid. These results indicate that fractionation-driven structural refinement is associated with enhanced neurochemical modulation in PC12 cells at the cellular level.

Overall, this work highlights the value of yellowfin tuna by-products as a marine protein resource and supports their potential utilization as a source of bioactive peptide ingredients. However, the present findings remain limited to in vitro peptide-fraction characterization and cell-based neurochemical readouts. Further validation in animal models, together with inhibitor-based or other mechanistic intervention studies, will be necessary before stronger conclusions can be drawn regarding physiological relevance or application potential. From an application perspective, TBP–MF may represent a promising marine-derived peptide fraction for future nutraceutical or functional-food development related to sleep support or broader neurofunctional support, although in vivo validation and sequence-specific studies remain necessary.

## Figures and Tables

**Table 2:** PeptideRanker scores and BIOPEP–UWM bioactivity motif profiling of candidate peptides from the TBP and TBP–MF groups.

Peptide Sequence (TBP)	PeptideRanker Score	Key Predicted Bioactivities (Active Motifs)	Peptide Sequence (TBP–MF)	PeptideRanker Score	Key Predicted Bioactivities (Active Motifs)
VVGLPGTR	0.302962	Antiamnestic (PG); Regulating (PG); ACE inh. (GLP, LPG); DPP–IV inh. (LP, VV)	DLATNPKPR	0.430282	ACE inh. (PR, LA); DPP–IV inh. (LA, KP)
MSTNPKPQR	0.291271	ACE inh. (PQR, TNP); DPP–IV inh. (KP, NP)	LSGQDLR	0.403348	Neuropeptide activity (GQ); Neuroprotective (LR); ACE inh. (GQ, SG); DPP–IV inh. (QD)
DLGEEHFK	0.19661	ACE inh. (GE, LG); DPP–IV inh. (EH, GE)	SGFQAEYVR	0.380427	ACE inh. (GF, SG); DPP–IV inh. (VR, AE)
FQDLVKDK	0.195682	ACE inh. (VK, FQ); DPP–IV inh. (FQ, LV)	HMADYELR	0.376066	Neuroprotective (LR); Regulating (DY); ACE inh. (DY, YE); DPP–IV inh. (MA, AD)
LSAEGADVR	0.188699	Neuropeptide activity (EG); Binding (EG); ACE inh. (GA, EG); DPP–IV inh. (GA, VR)	ALDVVGLR	0.356781	Neuroprotective (LR); ACE inh. (VG, GL); DPP–IV inh. (VV, AL)
SLLSGYDNK	0.171974	Regulating (SL); ACE inh. (GY, SG); DPP–IV inh. (LL, SL)	GILFVGSSR	0.339825	Neuropeptide activity (IL); ACE inh. (LF, VG); DPP–IV inh. (GI, IL)
HGDLGNVTK	0.154972	ACE inh. (HG, LG); DPP–IV inh. (NV, TK)	GLSDGEWQQ	0.257559	ACE inh. (GL, GE); DPP–IV inh. (GL, WQ)
AAEGVLTK	0.139185	Neuropeptide activity (EG); Hypotensive (AA); ACE inh. (AA, GV); DPP–IV inh. (AA, AE)	VTVPAYFNK	0.241983	ACE inh. (AY, VP); DPP–IV inh. (PA, VP)
VLGGQYVTR	0.135662	Neuropeptide activity (GQ); ACE inh. (GQ, GG); DPP–IV inh. (GG, QY)	TQEFIDR	0.200373	ACE inh. (TQ, EF); DPP–IV inh. (DR, QE)
VNQAEQLR	0.128261	Neuroprotective (LR); ACE inh. (LR); DPP–IV inh. (AE, NQ)	QQEALDKK	0.130172	ACE inh. (EA, KK); DPP–IV inh. (AL, KK)
TIANSDR	0.0832161	ACE inh. (IA, DR); DPP–IV inh. (IA, DR)	QVVDSHVR	0.116024	ACE inh. (VR, VV); DPP–IV inh. (VV, VR)
SVSEELTK	0.0802248	DPP–IV inh. (LT, SV)	TGADVVVTR	0.108484	ACE inh. (GA, TG); DPP–IV inh. (VV, GA)
LNVQAAAK	0.0776645	Hypotensive (AA); ACE inh. (AA, LN); DPP–IV inh. (AA, LN)	VTSGDTTR	0.0759831	ACE inh. (SG, GD); DPP–IV inh. (TR, TS)
GQVEVTGSK	0.0699837	Neuropeptide activity (GQ); ACE inh. (GS, GQ); DPP–IV inh. (EV, QV)	LTTDGKVR	0.06769	ACE inh. (GK, VR); DPP–IV inh. (VR, KV)
TTATDDVK	0.0346048	Binding (TAT); ACE inh. (VK); DPP–IV inh. (TA, AT)	VGNAVSEVK	0.0603922	ACE inh. (VK, VG); DPP–IV inh. (AV, EV)

Note: PeptideRanker scores indicate the relative predicted likelihood of peptide bioactivity based on sequence features; they do not represent homology-identification confidence or experimental validation. ACE inh., angiotensin-converting enzyme-inhibitory activity; DPP-IV inh., dipeptidyl peptidase IV-inhibitory activity. BIOPEP-derived functional labels indicate motif-level annotation matches rather than experimentally validated activities of the full peptide sequences.

## Data Availability

The raw data supporting the conclusions of this article will be made available by the corresponding authors on request.

## Abbreviations

The following abbreviations are used in this manuscript:

TBP	Tuna blood-meat peptide fraction
TBP–MF	Enriched major fraction of TBP obtained by Sephadex G-25 fractionation
TPH2	Tryptophan hydroxylase 2
GCH1	GTP cyclohydrolase 1
GABA	γ-aminobutyric acid
5-HT	5-hydroxytryptamine
BH4	Tetrahydrobiopterin
ACE	Angiotensin-converting enzyme
DPP-IV	Dipeptidyl peptidase IV
DH	Degree of hydrolysis
SEM	Scanning electron microscopy
DLS	Dynamic light scattering
DSC	Differential scanning calorimetry
DTG	Derivative thermogravimetry
FTIR	Fourier transform infrared spectroscopy
TGA	Thermogravimetric analysis
TG	Thermogravimetric curve
